# Culturomics Discloses Anti-Tubercular Enterococci Exclusive of Pulmonary Tuberculosis: A Preliminary Report

**DOI:** 10.3390/microorganisms8101544

**Published:** 2020-10-07

**Authors:** Mustapha Fellag, Nina Gouba, Marielle Bedotto, Moussa Sakana, Dezemon Zingué, Zékiba Tarnagda, Matthieu Million, Michel Drancourt

**Affiliations:** 1 IHU Méditerranée Infection, 13005 Marseille, France; mus.fellag@gmail.com; 2 Aix-Marseille Univ., IRD, MEPHI, IHU Méditerranée Infection, 13005 Marseille, France; marielle.bedotto@gmail.com (M.B.); matthieumillion@gmail.com (M.M.); 3 UFR-ST Département Sciences Biologiques, Université Nazi Boni, Bobo-Dioulasso 01 BP 1091, Burkina Faso; goubanina@yahoo.fr; 4 Institut de Recherche en Sciences de la Santé, Direction Régionale de l’Ouest, Bobo Dioulasso 01 BP 545, Burkina Faso; sakanafils@gmail.com (M.S.); zekiba@hotmail.com (Z.T.); 5 Centre Muraz, Bobo-Doulasso, Institut National de Santé Publique, Bobo-Dioulasso 01 BP 390, Burkina Faso; zinguedezemon@yahoo.fr

**Keywords:** gut microbiota, tuberculosis, *Mycobacterium tuberculosis*, *Mycobacterium bovis*, *Mycobacterium canettii*, *Enterococcus mundtii*, *Enterococcus casseliflavus*

## Abstract

*Mycobacterium tuberculosis* causes pulmonary tuberculosis, a deadly infection of which the clinical expression and prognosis are not fully understood at the individual level, apart from genetic susceptibility traits. We investigated whether individual gut microbiota may correlate with pulmonary tuberculosis status. Culturomics investigations of gut microbiota in two pulmonary tuberculosis patients and two controls in Burkina Faso found 60 different bacterial species in patients and 97 in controls, including 45 in common. Further analysis of the results at the individual level indicated seven bacteria, including *Enterococcus mundtii* and *Enterococcus casseliflavus*, which were exclusively cultured in controls. Blind quantitative PCR-based exploration of faeces samples in two cohorts in Burkina Faso and in France confirmed a nonsignificant association of *E. mundtii* and *E. casseliflavus* with controls. Further in vitro explorations found four *E. mundtii* and *E. casseliflavus* strains inhibiting the growth of *M. tuberculosis* strains representative of four different lineages as well as *Mycobacterium africanum*, *Mycobacterium canettii*, and *Mycobacterium bovis*, in an inoculum-dependent manner. Heat-killed *E. mundtii* or *E. casseliflavus* were ineffective. These unprecedented observations of direct interactions between gut *E. mundtii* and *E. casseliflavus* with *M. tuberculosis* complex mycobacteria suggest that gut microbiota may modulate the expression of pulmonary tuberculosis.

## 1. Introduction

Tuberculosis is a deadly bacterial infection due to mycobacteria forming the *Mycobacterium tuberculosis* complex (MTBC), and is responsible for a high burden of morbidity and mortality in the most countries worldwide [1]. In 2018, the World Health Organization (WHO) reported 10 million new cases and 1.5 million deaths [2].

The interplay between host and pathogen determining the clinical expression and prognosis of tuberculosis is not fully understood at the individual level. Indisputably, the *M. tuberculosis* inoculum, genotype and antibiotic susceptibility profile may play in part the clinical expression and prognosis of pulmonary tuberculosis [3,4]. Host traits mostly related to genetic alterations of the interferon gamma pathway have also been elucidated in highly susceptible tuberculosis individuals and families [5]. Such genetic traits have been significantly associated with certain clinical expressions of tuberculosis, such as onset in the early years of life [6,7]. Accordingly, at the population level, discrete co-evolution of certain *M. tuberculosis* genotypes with certain populations has been clearly established in some geographic areas [3,7,8,9].

However, the factors mention above may not explain all the variations observed in the clinical spectrum of tuberculosis and the hypothesis that individual microbiota may also govern part of the clinical expression of tuberculosis, has emerged [10]. A few studies recently indicated significant correlations between the repertoire of microorganisms in the digestive tract microbiota and pulmonary tuberculosis [11,12,13], yet these studies relied upon metagenomics, leaving it unknown whether living organisms were indeed significantly associated with tuberculosis.

In order to advance this knowledge, we herein embarked on a preliminary study of the digestive tract microbiota in individuals with and without pulmonary tuberculosis using culturomics, in order to discover whether some living microorganisms detected in the digestive tract microbiota, including bacteria and archaea, were significantly correlated and anticorrelated with pulmonary tuberculosis prior to anti-TB treatment.

## 2. Materials and Methods

### 2.1. Ethics Statement and Population Information

A total of 150 stool samples collected from 150 individuals were included in this study. Fifty stools specimens from fifty individuals were collected as part of a tuberculosis diagnosis kit in patients being routinely investigated for medical suspicion of pulmonary tuberculosis in the IHU Méditerranée Infection, Marseille, France [14]. Microbiological diagnosis using sputum Ziehl-Neelsen staining, culture, and molecular biology (GeneXpert) identified 14 samples belonging to tuberculosis patients and 36 belonging to non-tuberculosis patients. Ethical approval was obtained from the Institutional Ethics Committee of the Institut Hospitalier Universitaire—Méditerranée Infection (Marseille, France) under number 2012-A01598-35. One hundred individuals were sampled in Burkina Faso. The participants were recruited in the tuberculosis Diagnosis and Treatment Centre at Bobo-Dioulasso. Among 100 samples collected in Burkina Faso, 50 were collected from patients with confirmed pulmonary tuberculosis and 50 from individuals without pulmonary tuberculosis based on microscopy test using sputum Ziehl-Neelsen staining. The ethics statement was approved by the Research Ethics Committee in Health of Science, Comité d’Éthique Institutionnel pour la Recherche en Science de la Santé (CEIRS) (N/Ref.002-2018-CEIRS) (Bobo-Dioulasso, Burkina Faso). After written consent was obtained from all participants for sample collection and subsequent analyses, stool and sputum samples were collected from patients and were placed in sterile containers. Stool and sputum samples were stored at −80 °C and sent for further analyses to the partner laboratory at the IHU at Marseille, France. All stool samples were collected prior to diagnosis and prior to starting treatment for tuberculosis.

### 2.2. Culturomics

To analyze the differences between the digestive microbiota of patients with pulmonary tuberculosis and subjects without pulmonary tuberculosis, culturomics was performed on 4 stool samples collected in Burkina Faso, 2 stool samples randomly selected from samples collected from individuals positive for pulmonary tuberculosis and 2 stool samples randomly selected from samples collected from individuals negative for pulmonary tuberculosis (Table 1).

The culture was performed as previously described with modifications [15]. In brief, one gram of stool was diluted into 1 mL of Dulbecco’s phosphate-buffered saline (DPBS 1X, Thermo Fisher Scientific, Illkirch, France) and the suspension was inoculated into each of the two blood culture bottles (BD Bactec Plus; Becton Dickinson, Le Pont-de-Claix, France), one for anaerobic culture and the other for aerobic culture. At the same time, one-gram stool suspensions were inoculated on YCFA medium modified for solid and liquid medium, prepared as described on the web site (https://www.dsmz.de/microorganisms/medium/pdf/DSMZ_Medium1611.pdf) (Deutsche Sammlung von Mikroorganismen und Zellkulturen GmbH, Braunschweig, Germany). The blood culture bottles and YCFA medium were previously supplemented with 3 mL of filtered rumen fluid and 3 mL of sheep blood (Becton Dickinson). The inoculated blood culture bottles and negative controls, represented by blood culture bottle inoculated with DPBS, were incubated at 37 °C. Inoculations on solid agar were performed at three hours, six hours, nine hours, twenty-four hours, three days, seven days, and 14 days post-incubation. For each inoculation, 1 mL of supernatant was collected and diluted in 900 µL of DPBS in serial dilutions from 1/10 to 1/10^10^. These dilutions were inoculated on 5% sheep blood agar (bioMérieux, La Balme-les-Grottes, France) incubated at 37°C for 48 h, respecting the strict atmospheric conditions of aerobic and anaerobic culture. All colonies were sub-cultured and identified by matrix-assisted laser desorption ionisation-time of flight mass spectrometry (MALDI-TOF-MS), as previously described [16]. Unidentified colonies by MALDI-TOF-MS were identified using 16S rRNA gene sequencing as previously described [17]. The bacterial species isolated from four stools of each person were compared by preparing a Venn diagram (http://bioinformatics.psb.ugent.be/webtools/Venn/).

### 2.3. Real-Time Quantitative PCR

In order to further explore the prevalence of two bacteria of interest (*E. casseliflavus* and *E. mundtii*) in the stools of TB-positive and TB-negative patients, 150 stools specimens were tested with molecular PCR-based assays as previously described [18]. For each sample, DNA were extracted from 200 mg of stool with a semi-automated method using an EZ1 DNA Tissue Kit (Qiagen, Hilden, Germany) and EZ1 Advanced XL extractor (Qiagen). Real-time qPCR reactions incorporated a total volume of 20 µL including 10 µL LightCycler^®^ 480 Probes Master (Roche Diagnostics GmbH, Mannheim, Germany), 0.5 µL of each primer, 0.5 µL of probe, 3 µL of distilled water, 0.5 µL of Uracil-DNA glycosylase (UDG) and 5 µL of DNA. Analyses were performed using the thermal cycler CFX96 Touch™ (BIO-RAD, Marnes-la-Coquette, France). DNA extraction, PCR inhibitors and total bacterial richness was measured using real-time PCR targeting the universal bacterial 16S rRNA gene in the presence of negative controls (Supplementary Table S1). Two quantitative PCR systems were designated for *Enterococcus casseliflavus* and *Enterococcus mundtii*. Finally, the presence of *Methanobrevibacter smithii* was detected using real-time PCR, as previously described [19] (Supplementary Table S1). qPCR results were interpreted as positive when the cycle threshold (Ct) value was less than or equal to 39.

### 2.4. Anti-Tuberculosis Activity Assays

The antituberculosis activity of seven bacteria specifically found in the gut microbiota of two tuberculosis-free patients was assessed using the agar well diffusion method, with modifications [20,21]. Bacteria were cultured on COS agar medium according to the conditions necessary for the growth of each bacterium. Fresh culture colonies were suspended in DPBS, adjusted to 15 McFarland and serial dilution was performed in DPBS. The anti-tuberculosis activity of these seven bacteria was then tested against eight MTBC clinical strains including *M. tuberculosis* lineage 1 strain (CSURP9458)*, M. tuberculosis* lineage 2 strain Beijing CSUR Q1316 [18] *M. tuberculosis* lineage 3 strain CSURP7204, *M. tuberculosis* lineage 4 strain CSUR P7739 [22], *M. africanum* strain CSURP7201, *M. canettii* strain CIPT140010059, and *M. bovis* strain CSURP7222 [23]. All strains were cultured in Middlebrook 7H10 culture medium and calibrated to 10^7^ CFU/mL, as previously described [24]. This suspension was inoculated in Middlebrook 7H10 agar with Middlebrook OADC enrichment (Becton Dickinson) at 37 °C for one day, in Petri dishes (Ø × H = 90 × 13 mm, Dutscher, 67170 Brumath, France) or in square Petri dish plates (W × L × H = 120 × 120 × 15 mm Dutscher) after 200 µL wells had been made using a sterile tip. Each bacterial suspension was then deposited in each well alongside sterile DPBS in the negative control well and the plates were incubated at 37 °C and observed every three days up to 15 days, to monitor and enumerate any zones of MTBC growth inhibition around the wells. To measure any potential pH changes in the wells, a 200 μL Middlebrook 7H10 agar disk was incubated for 48 h at 37 °C with a 10 MacFarland suspension of each bacterial species (after growth on Middlebrook 7H10 agar had been verified); and the pH was measured. In order to further assess the potential *M. tuberculosis* growth inhibition by dead bacteria, the bacterial suspensions were inactivated by one-hour incubation at 100°C and the potential inhibitory effect of inactivated bacteria was tested as described above. All these manipulations were carried out three times in the presence of DPBS as a negative control.

### 2.5. Statistical Analyses

Data was entered into Microsoft Excel for Office 365 to calculate median, average and standard deviation. A clustering heatmap was used to visualise the potential clustering of cultured gut bacterial repertoires in positive and negative pulmonary tuberculosis patients. The heatmap and the principal component analysis were performed using XLSTAT v2019.1 statistical and data analysis solution (Long Island, NY, USA (https://www.xlstat.com). Statistical tests were performed on the website biostaTGV (https://biostatgv.sentiweb.frS) including Student’s T test to compare DPBS and the bacteria inhibition diameter and the ANOVA test was used to compare the susceptibility of mycobacteria to enterococci. Differences were considered to be statistically significant when *p* < 0.05.

## 3. Results

### 3.1. Bacterial Culturomics

**Figure 1:**
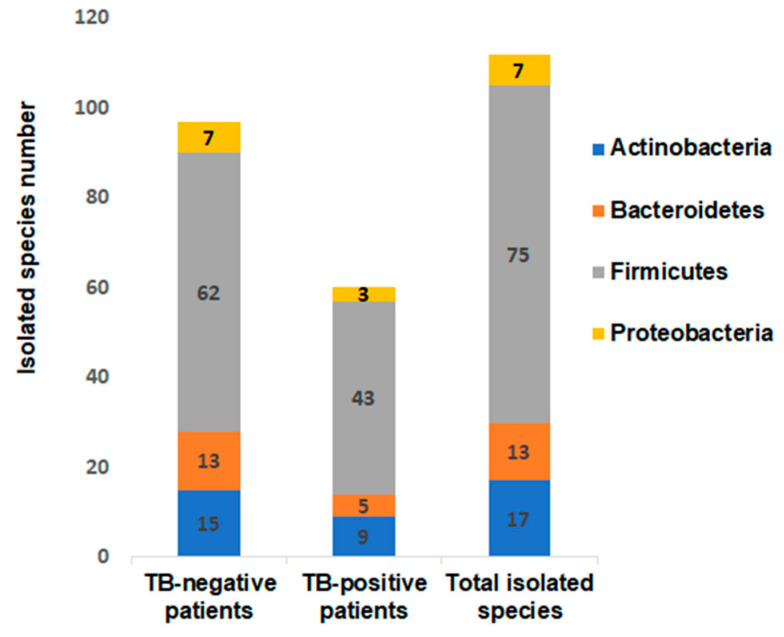
Cultured gut bacteria distribution between tuberculous and nontuberculous patients. Phyla and number of isolated bacterial species per group included tuberculosis patients and controls.

We investigated faeces samples collected from two TB-positive and two TB-negative patients in Burkina Faso. The MALDI-TOF MS identification yielded a total of 112 different bacteria species, divided into 49 genera and 34 families including one unclassified family and four phyla *Actinobacteria, Bacteroides, Firmicutes* and *Proteobacteria*. A total of 60 bacterial species scattered across 28 genera were identified in the stools collected from two TB-positive patients and 97 species scattered across 43 genera were identified in the stools collected in two TB-negative patients. At the level of bacterial phyla, Firmicutes were more prevalent in the TB-positive patients, whereas Bacteroidetes were more prevalent in the TB-negative patients (Figure 1).

**Figure 2:**
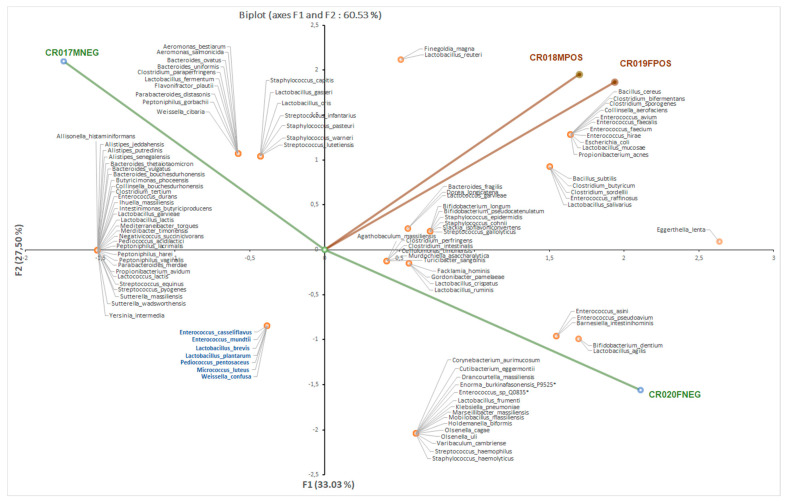
Principal component analysis (PCA) of 112 bacterial species isolated from the guts of the four individuals included in this study. PCA revealed a clustering of bacterial composition in the tuberculosis patients between the bacteria isolated in the two tuberculosis patients.

At the level of bacterial genus, culturomics performed for four individuals showed that the *Clostridium* bacteria were more abundant in the TB-positive patients (seven in 28; 25%) than in the TB-negative patients (four in 43; 9%). Furthermore, we observed a reduction in the number of *Bacteroides, Enterococcus, Streptococcus* and *Staphylococcus* members in the TB-positive patients compared to the TB-negative patients and saw that the number of bacterial species was lower in the TB-positive patients. Preparing out a heatmap (Supplementary Figure S1) and a principal component analysis (Figure 2) showed a clustering of the bacteria isolated from the two TB patients.

**Figure 3:**
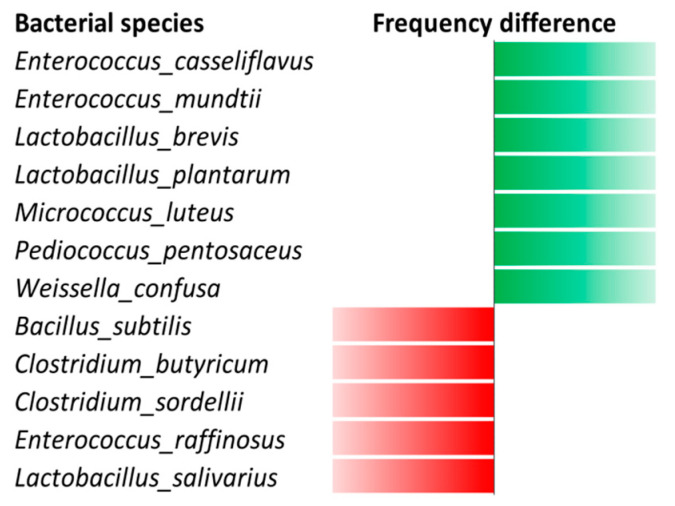
Frequency analysis performed on bacteria isolated from the four individuals reveals seven bacteria isolated only in the non-tuberculous patients shown in green and five bacteria isolated only in the tuberculous patients shown in red.

The Venn diagram highlighted 15 bacterial species isolated only in the TB-positive patients, 52 bacterial species isolated only in TB-negative patients and 45 bacterial species that were isolated in both the TB-positive and TB-negative patients (Supplementary Figure S2). More precisely, seven bacterial species (*Enterococcus casseliflavus*, *Enterococcus mundtii*, *Lactobacillus brevis*, *Lactobacillus plantarum*, *Micrococcus luteus*, *Pediococcus pentosaceus*, and *Weissella confusa*) were cultured in both the two TB-negative patients and were not cultured in both the two TB-positive patients, while five bacterial species (*Bacillus subtilis*, *Clostridium butyricum*, *Clostridium sordellii*, *Enterococcus raffinosus* and *Lactobacillus salivarius*) were shared by the two TB-positive patients and were absent from the two TB-negative patients (Figure 3). *M. smithii* was detected by qPCR in all four of these stool samples. Finally, two new species were isolated from one TB-negative patient [25,26,27].

### 3.2. Real-Time PCR Assays

In order to further explore the prevalence of *E. casseliflavus* and *E. mundtii* in the faeces of TB-positive and TB-negative patients, 50 stools were collected from 50 patients being routinely investigated for medical suspicion of pulmonary tuberculosis in the IHU Méditerranée Infection, Marseille, France [28] and 100 specimens were collected from 100 patients routinely investigated for medical suspicion of pulmonary tuberculosis in Burkina Faso (97 specimens collected from individuals in Bobo-Dioulasso and three from individuals in Houndé). In the presence of negative controls (which remained negative), quantitative PCR analysis targeting the 16S rRNA gene of all bacteria within these 150 faeces samples yielded a median Ct of 18.44 and an average Ct average of 18.32 (Ct standard deviation, 3.08; range 11.5–24.4). In the subset of 64 TB-positive patients, the same analysis yielded a median Ct of 18.18 and an average Ct of 18.31 (Ct standard deviation, 2.62; range 13.5–23.8). In the subset of 86 TB-negative patients, the same analysis yielded a median Ct of 17.5 and an average Ct of 18.32 (Ct standard deviation, 3.39; range 11.5–24.4). There were no significant differences in the Ct values between TB-positive and TB-negative patients, indicating an absence of PCR inhibitors in any of the faeces samples investigated here. This allowed us to further investigate the negative results in further quantitative real-time PCR assays, regardless of the TB status of the patient. *M. smithii* was detected by qPCR in 139/150 (92.7%) faeces samples: five faeces collected from TB-positive patients and six from TB-negative patients were negative. Accordingly, in the very same 150 individuals, blind quantitative real-time PCR targeting *E. mundtii* yielded 28 (18.22%) positive individuals with a median Ct of 35.47, an average Ct of 34.8, and Ct standard deviation of 2.61, including nine out of 64 (14%) TB-positive patients and 19 in 86 (22%) TB-negative patients (*p* = 0.21). All 28 *E. mundtii*-positive faeces specimens had been collected from Burkinabe individuals and none from French individuals. In TB-positive patients, *E. mundtii* was detected with a median Ct of 34.5, an average Ct of 34.2, and standard deviation of Ct 2.5 (Ct value range, 30.6–38.8), no different from Ct values obtained in TB-negative patients with, respectively, a median Ct of 34.48, an average Ct of 34.23, and standard deviation of Ct 2.6 (Ct value range, 28.8–38.44). Quantitative real-time PCR targeting *E. casseliflavus* yielded 78 in 150 (52%) positive individuals with a median Ct of 34.8, an average Ct of 34.1, and a Ct standard deviation of 2.85, including 28 in 64 (43%) TB-positive patients and 50 in 86 (58%) TB-negative patients (*p* = 0.08). In TB-positive patients, *E. casseliflavus* was detected with a median Ct of 34.45, an average Ct of 35.06, and a standard deviation Ct 2.06 (Ct value range, 28.44–37.19), no different from Ct values obtained in TB-negative patients with, respectively, a median Ct of 34.11, an average Ct of 34.9, and a standard deviation Ct 2.85 (Ct value range, 22.67–38.85).

### 3.3. Antitubercular Activity

**Figure 4:**
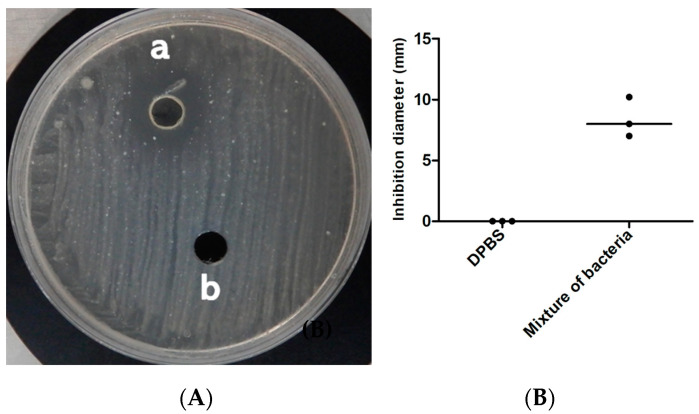
Effect of mixture bacteria on growth of *M. tuberculosis* lineage 2. (**A**) Observation of the inhibition spectrum caused by the mixture bacteria (a) on *M. tuberculosis* and DPBS (b) as negative controls. The picture was prepared 12 days after incubation of *M. tuberculosis* at 37 °C. (**B**) The inhibition test was carried out three times, the diameter of inhibition was measured on the three cultures. The image indicates the median.

**Figure 5:**
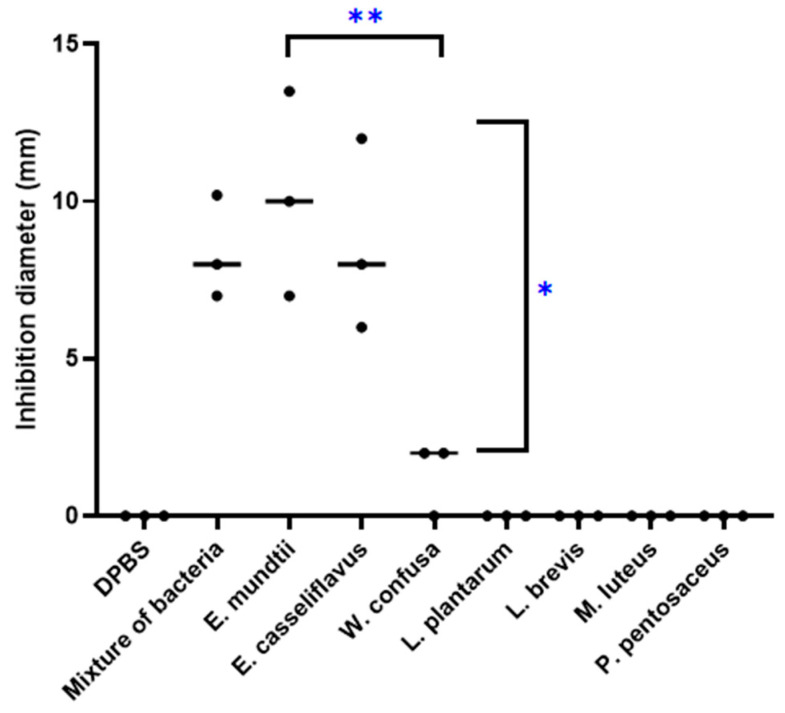
In vitro activity effect of the seven bacteria isolated only from non-tuberculosis patients against *M. tuberculosis* lineage 2. For each bacterium and DPBS negative control tests were carried out in triplicate. “**” Comparison of the mean inhibition diameter of *M. tuberculosis* by *E. mundtii* vs. the mean diameter inhibition of *M. tuberculosis* by *W. confusa* (*p* Value = 0.0156). “*” Comparison of the mean inhibition diameter of *M. tuberculosis* by *E. casseliflavus* vs. the mean diameter inhibition of *M. tuberculosis* by *W. confusa* (*p* Value = 0.0345). Means inhibition diameters were compared using Tukey’s test (*p* < 0.05).

**Figure 6:**
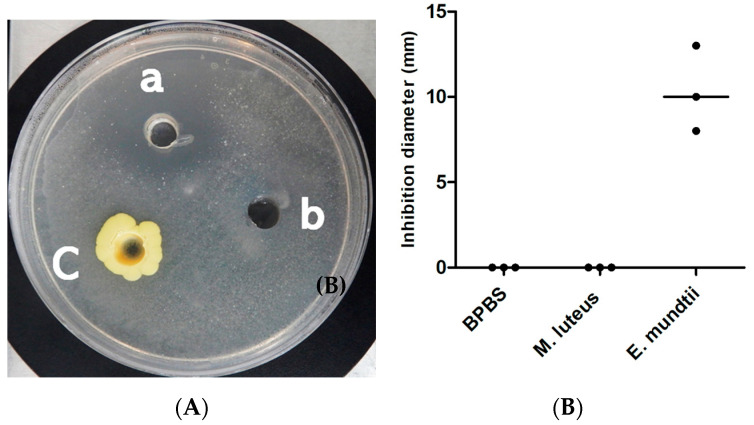
In vitro activity of *E. mundtii* against *M. tuberculosis* lineage 2 growth. (**A**) Picture showing the inhibitory effect of *E. mundtii* (a) on *M. tuberculosis*, DPBS buffer (b) and *M. luteus* (c) as a negative control. (**B**) Triplicate test inhibition of *E. mundtii* against *M. tuberculosis*.

The mixture of these seven bacteria lowered the local pH of the Middlebrook culture medium to 5 and inhibited growth of the *M. tuberculosis* strain Beijing CSUR Q1316 (Figure 4). Further experiments aimed to determine which of these seven bacteria were individually inducing the inhibition of growth of *M. tuberculosis*, first indicated that the local pH of the Middlebrook culture medium varied from pH 5 for *Lactobacillus* species to pH 6 for *M. tuberculosis* and pH 7 for *M. luteus*. Further, inhibition of growing *M. tuberculosis* was reproducibly observed only for *E. mundtii* and *E. casseliflavus* but not for *Lactobacillus brevis, Lactobacillus plantarum, Micrococcus luteus, Pediococcus pentosaceus* and *W. confusa* (Figure 5). Inhibition growth of *M. tuberculosis* was observed for *E. mundtii* and *E. casseliflavus* with bacterial inoculum of 15 McFarland but not for inoculum of 5 McFarland. (data not shown). The *M. tuberculosis* growth inhibition effect was observed on all tested strains of the MTBC including *Mycobacterium canettii*, *Mycobacterium bovis* and *Mycobacterium africanum* with diameters of growth inhibition being on average three times larger for *M. canettii* than for the other species (Table 2) (Figure 6). *E. mundtii* and *E. casseliflavus* have a similar inhibitory effect against all tested MTBC, except for *M. canettii* which is significantly more susceptible than the other species of the MTBC (*p* = 2.14 × 10^−7^). The *M. tuberculosis* growth inhibition effect was not merely due to changes in the pH, as the pH of the suspension was increased from 5 to 6, however *M. tuberculosis* also reduced the pH of the suspension to 6. However, a few colonies growing in the *M. tuberculosis* growth inhibition zone around either *E. mundtii* or *E. casseliflavus* were confirmed to be colonies of *M. tuberculosis* by whole genome sequencing. Genome sequences have been deposited in the European Molecular Biology Laboratory (EMBL).

## 4. Discussion

Previous metagenomics studies all indicated that the repertoire of bacteria in the gut microbiota significantly differed between pulmonary tuberculosis patients and controls, suggesting biological relationships between pulmonary tuberculosis and gut microbiota [10]. However, contradictory data from these studies complicated the interpretation of these differences. Decreases in the number of bacterial species and bacterial diversity reported in TB-positive patients [29] including children [30], contrasted with the reported increased diversity in TB-patients in another study [12]. In children, microbial richness was not found to significantly differ between TB-positive children and controls, although the Simpson index was found to be significantly lower in TB-positive children [30] with five different bacterial species found to be significantly balanced between the two groups [30]. The interpretation of metagenomics studies data was limited by the inherent profounder bias of the method, lack of accurate bacterial species identification [10], and the fact that detected organisms may not be viable, thus rendering them to be of limited physiological interest.

In this study, we used culturomics (in addition to PCR-based detection of the methanogen *M. smithii*) to overcome some limitations of the metagenomics studies, and confirmed the decreased prevalence in Bacteroidetes in TB-positive patients compared to TB-negative ones [12] and the increase of *Clostridium* members. This was in line with observations made in experimental severe tuberculosis in macaques [31]. Unexpectedly, culturomics yielded two Firmicutes *E. casseliflavus* and *E. mundtii* exclusively in the faeces of TB-negative patients. These two Firmicutes were further qPCR-detected with an increased prevalence in the faeces of TB-negative patients in two independent cohorts. These observations were extended by experimental observations showing these two Firmicutes inhibited the growth of *M. canettii*, regarded as the MTBC species most closely related to the common ancestor of the complex and a pathogen responsible for tuberculosis in exposed individuals in the Horn of Africa [1], and that of *M. tuberculosis* and *M. bovis* responsible for tuberculosis in humans and animals [32]. These observations, all reproduced at least three times, were attested to by the negativity of the negative controls. We also observed that heat-killed bacteria no longer inhibited MTBC growth which was inoculum-dependent and not merely related to pH variation. All observations suggest an active process which remains to be elucidated. The two Firmicutes are known to produce bacteriocins, yet none of these bacteriocins has ever been demonstrated to be active against *M. tuberculosis* [33,34,35]. As for *E. mundtii*, all previous studies have dealt with the antibiotic activity of environmental isolates against opportunistic pathogens, mainly *Listeria monocytogenes*, such as *E. mundtii* fish isolates LP17 and LP18 [36]. Identifying the mechanism of action of *E. mundtii* and *E. casseliflavus* against MTBC was beyond the scope of the present study, yet the *E. mundtii* and *E. casseliflavus* strains here investigated all encode for bacteriocin, as anticipated by their whole genome sequence.

Interactions between *M. tuberculosis* and inhibitory enterococci are likely to occur in the gut. *M. tuberculosis* is routinely cultured from the faeces collected from TB-positive patients but not from TB-negative ones [28]. In the “Lübeck Disaster 1929–1933”, oral inoculation of the BCG vaccine inadvertently contaminated 251 neonates with *M. tuberculosis*, causing clinical tuberculosis in 228 (90.83%) cases, while five in 251 (1.99%) of the children died of unrelated causes. Although 18 neonates showed no signs of tuberculosis, 72 died. Follow-up of the 174 surviving children revealed only six sick children, while the others’ symptoms were spontaneously resolved. Such a heterogeneous natural history of tuberculosis in neonates in the Lübeck series of cases may partly be explained by different gut microbiota maturation in the neonates [37]. Indeed, translocating *M. canettii* and *M. tuberculosis* has been shown to lead to both disseminating in the lymphatic and lung tissues after experimental gavage in mice [18,38]. It would be interesting to further document *E. mundtii* and *E. casseliflavus* interactions with *M. tuberculosis* in a mouse model, to observe interference with *M. tuberculosis* gut survival, translocation, and recirculation in the lungs. Complementary, indirect interactions may be mediated by inflammation and immune cells in the exact model of what is now established in immune cancerology [39]. As an example, gut *Prevotella*, the prevalence of which was increased in TB-positive children and adults [12,30], were shown to correlate with blood CD4-positive lymphocytes and have been suggested to boost the inflammatory response to TB [12]. Likewise, *Clostridium* members that were found to be more abundant in TB-positive patients, induce Treg production in mice guts and an increased number of Venus+CD4+ cells in the liver and lungs [40]. *Clostridium* members are short-chain fatty acid (SCFA) producers inducing anti-inflammatory production (IL-10) [41], and SCFAs metabolites are acknowledged to modulate immune and proinflammatory responses to pulmonary TB [42].

This is a preliminary study involving by culturomics only in four individuals, so that the generalized of data here reported are not possible today and will required additional studies in larger population. Also, this study disclosed potential limitations in the enterococci interactions with *M. tuberculosis*. First, *E. mundtii* was detected only in faeces samples collected from Burkinabe individuals and not from French ones, suggesting that gut carriage of *E. mundtii* is population-dependant and is probably linked to diet, given that *E. mundtii* has been found in several plants and meat entering the human food chain [43,44]. Second, we did observe a few *M. tuberculosis* colonies that were resistant to *E. mundtii*-inhibition, suggesting that the mechanism of growth inhibition is not absolute in one *M. tuberculosis* population.

## 5. Conclusions

In conclusion, the data reported here indicate a higher bacterial diversity in non-tuberculous individuals versus pulmonary tuberculosis patients. In addition, we confirm a protective effect of certain gut microbiota and, particularly, of the Firmicutes *E. casseliflavus* and *E. mundtii* against pulmonary tuberculosis. Further studies may evaluate the relative recombination of innate immunity in these observations such as macrophage and cytokine expression induced by enterococci and *E. mundtii*, in particular during the intracellular phase of tuberculosis. Depending on the elucidation of the underlying mechanisms and topography of the inhibition and extent of *M. tuberculosis* complex, some of these Firmicutes may be considered as probiotic adjuvants to conventional antibiotics in the fight against deadly tuberculosis.

## Figures and Tables

**Table 1:** Characteristics of the four individuals studied by culturomics.

Patients	Sex	Age	Weight	Size	BMI	Smear Result	Sputum Culture	GeneXpert
CR017	M	45	90	1.81	27	negative	negative	negative
CR018	M	65	56	1.68	19	positive+++	positive	positive
CR019	F	28	50	1.60	19.53	positive+++	positive	positive
CR020	F	22	73	1.66	26.54	negative	negative	negative

**Table 2:** Inhibition effect of *E. mundtii* and *E. casseliflavus against M. tuberculosis* complex growth, DPBS as a negative control.

MTBC Strain	DPBS	Mixture	*E. mundtii*	*p*-Value *	*E. casseliflavus*	*p*-Value *
***M. tuberculosis* L 2**	0	8.4 ± 1.2	10.83 ± 1.77	0.015	9.1 ± 1.88	0.023
***M. tuberculosis* L1**	0	-	10.66 ± 3.40	0.032	9.83 ± 1.75	0.0104
***M. tuberculosis* L 3**	0	-	8.16 ± 1.04	0.005	6.33 ± 1.52	0.01884
***M. tuberculosis* L 4**	0	-	9.66 ± 3.05	0.031	8.83 ± 3	0.000516
***M. africanum***	0	-	9.66 ± 1.52	0.008	8.33 ± 1.52	0.011015
***M. canettii***	0	-	29.33 ± 1.15	0.0005	27.33 ± 2.08	0.00192
***M. bovis***	0	-	11.33 ± 2.08	0.011	10.66 ± 2.51	0.0180

* *p* value was obtained using student test. Experiments were triplicate. The growth inhibition diameters are expressed in mm. Values are expressed as mean ± standard deviation.

## Supplementary Materials

The following are available online at https://www.mdpi.com/2076-2607/8/10/1544/s1. Figure S1: Venn diagram analysis perform on the bacterial species isolated in the two groups (tuberculous and nontuberculous patients) showing the differential distribution and the common bacteria, Figure S2: Heatmap clustering of bacterial species cultured in the four individuals included in this study showing the bacterial species distribution between tuberculous and nontuberculous patients. Red and green scores indicate the absence and presence of bacterial species, respectively in tuberculosis patients (CR019FPOS and CR018MPOS) and control individuals (CR020FNEG and CR017MNEG), Table S1: Primers and probes used in this study.
