# Supplementary material for: Culturomics Discloses Anti-Tubercular Enterococci Exclusive of Pulmonary Tuberculosis: A Preliminary Report
Source: Microorganisms. 2020 Oct 7;8(10):1544. doi: 10.3390/microorganisms8101544 (PMC7599504; doi:10.3390/microorganisms8101544)
Supplement: Supplementary file 1 [file microorganisms-08-01544-s001.zip › fichires S/S Table 1 .docx]

**Table S1.** Primers and probes used in this study

| Organism | Target region | Name | Primers/probes |
| --- | --- | --- | --- |
| All bacteria | 16S rRNA | TTB_16S_F  TTB_16S_R  TTB338K_P | 5′-AGAGTTTGATCMTGGCTCAG-3′  5′- TTACCGCGGCKGCTGGCAC-3′  5′-FAM-CCAKACTCCTACGGGAGGCAGCAG-3′ |
| *Methanobrevibacter smithii* | 16S rRNA | Smit_6S-740F  Smit_16S-862R  Smit.16S_P | 5′-CCGGGTATCTAATCCGGTTC-3′  5′-CTCCCAGGGTAGAGGTGAAA-3′  5′- FAM-CCGTCAGAATCGTTCCAGTCAG-3′ |
| *Enterococcus casseliflavus* | hyp.p | EC_hypp_F  EC_hypp_R  EC_hypp_P | 5′-CAGTAAAATACAACGACCACAAACC-3′  5′-GGTAGCACTCCATAAATGACAGATAC-3′  5′-FAM-GCGCCGAGTATCATGGGGCC-3′ |
| *Enterococcus mundtii* | rpoB | Em_rpoB_F  Em_rpoB_R  Em_rpoB_P | 5′-ACCTGAATCCTTCCGCGTAT-3′  5′-TGTGATCAAATCATCGTCTTCG-3′  5′-FAM-CAGCGAGATCGAACTTCGCGACAT-3′ |
