# Supplementary figures and images for: Culturomics Discloses Anti-Tubercular Enterococci Exclusive of Pulmonary Tuberculosis: A Preliminary Report
Source: Microorganisms. 2020 Oct 7;8(10):1544. doi: 10.3390/microorganisms8101544 (PMC7599504; doi:10.3390/microorganisms8101544)

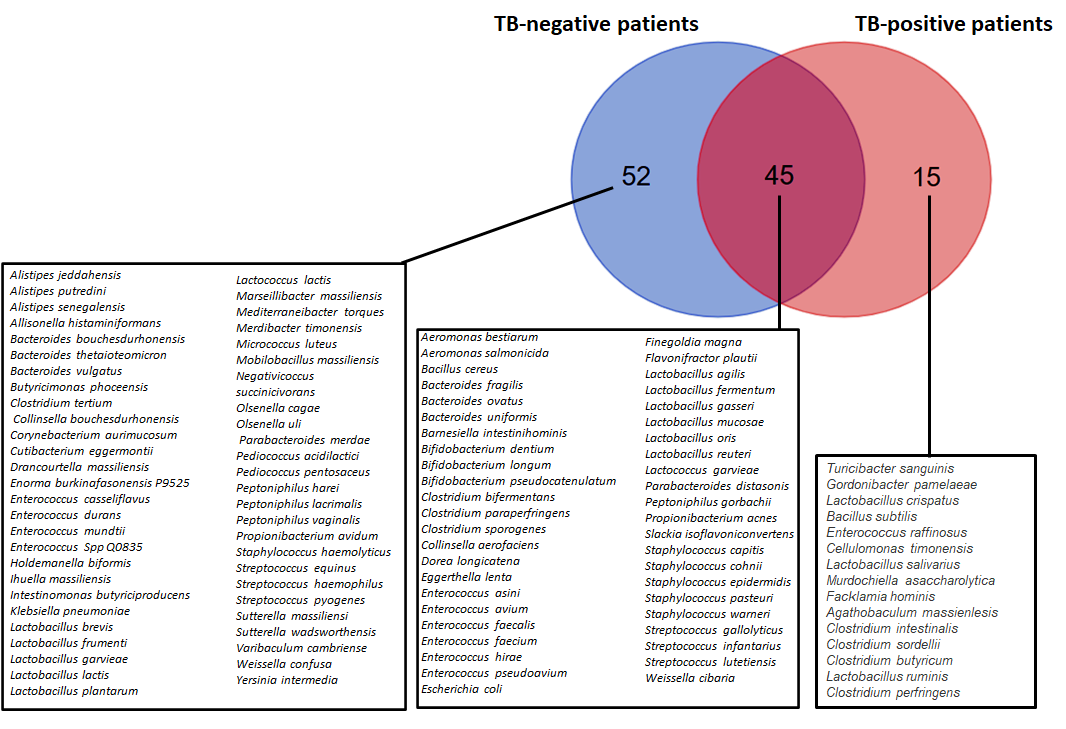

Supplement: Supplementary file 1 [file microorganisms-08-01544-s001.zip › fichires S/S1 Fig300.tiff]

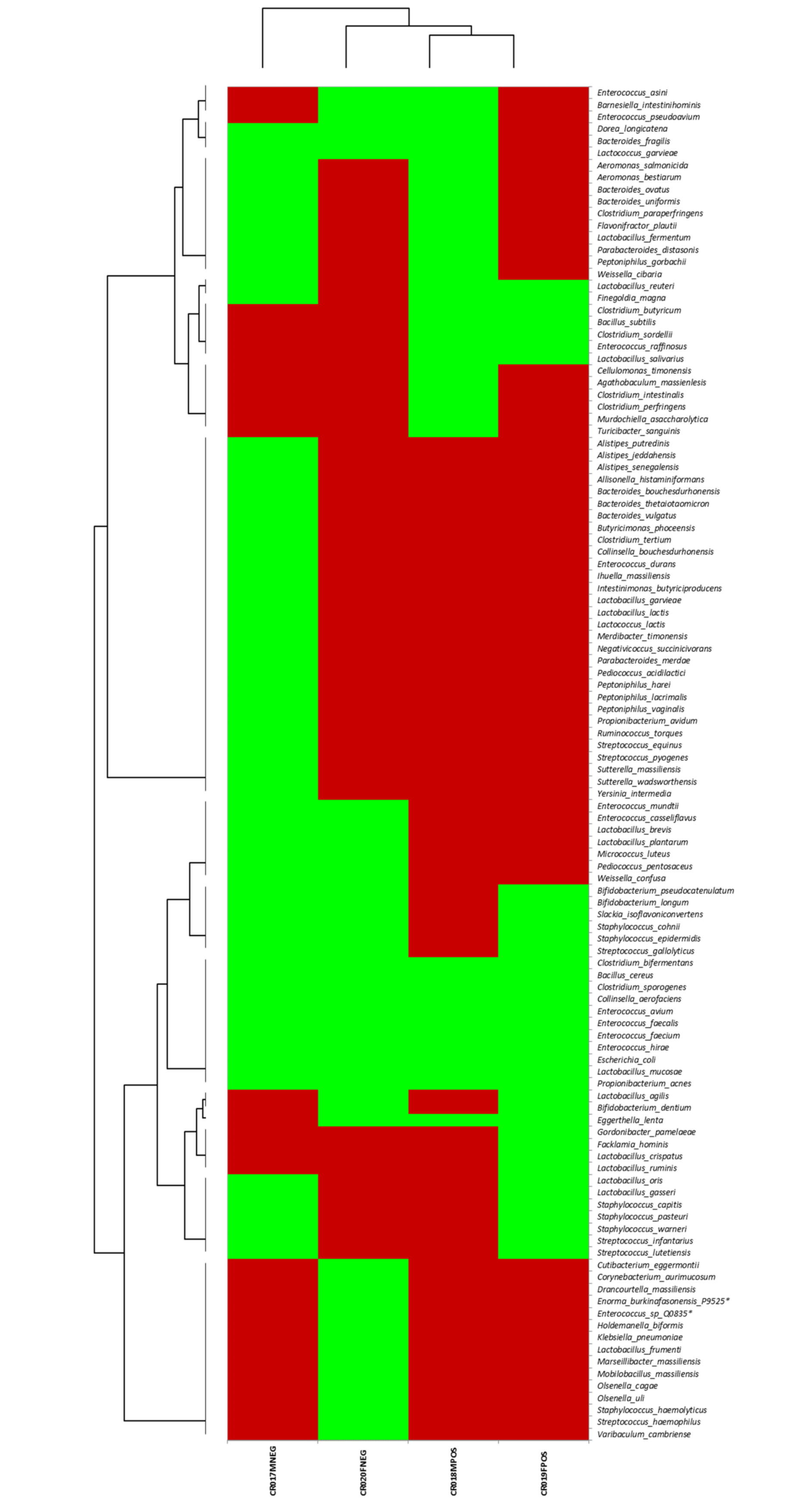

Supplement: Supplementary file 1 [file microorganisms-08-01544-s001.zip › fichires S/S2 Fig300.tiff]
